# Socioeconomic and demographic risk factors in COVID-19 hospitalization among immigrants and ethnic minorities

**DOI:** 10.1093/eurpub/ckab186

**Published:** 2021-10-27

**Authors:** Sabrina Islamoska, Jørgen Holm Petersen, Thomas Benfield, Marie Norredam

**Affiliations:** 1 Danish Research Centre for Migration, Ethnicity and Health, Section of Health Services Research, Department of Public Health, University of Copenhagen, Copenhagen, Denmark; 2 Department of Infectious Diseases, Copenhagen University Hospital—Amager and Hvidovre, Hvidovre, Denmark; 3 Institute of Clinical Medicine, Faculty of Health and Medical Sciences, University of Copenhagen, Copenhagen, Denmark

## Abstract

**Background:**

Immigrants and ethnic minorities have been shown to be at increased risk of hospitalization from COVID-19. Our aim was to analyse the contribution of socioeconomic and demographic risk factors on hospital admissions for COVID-19 among immigrants and ethnic minorities compared to the majority population.

**Methods:**

We used nationwide register data on all hospitalized COVID-19 cases between February and June 2020 (*N* = 2232) and random controls from the general population (*N* = 498 117). We performed logistic regression analyses and adjusted for age, sex, comorbidity, and socioeconomic and demographic factors. The main outcome measure was hospitalization with COVID-19 and was estimated using odds ratios (OR) and 95% confidence intervals (95% CI).

**Results:**

Among 2232 COVID-19 cases, the OR of hospitalization with COVID-19 among immigrants and descendants of non-Western origin was 2.5 times higher (95% CI: 2.23–2.89) compared with individuals of Danish origin with most pronounced results among individuals from Iraq, Morocco, Pakistan and Somalia. The OR was largely attributed to comorbidity and socioeconomic factors, especially household size, occupation, and population density.

**Conclusion:**

There is a significantly higher OR of hospitalization with COVID-19 among non-Western immigrants and ethnic minorities compared with ethnic Danes. This knowledge is crucial for health policymakers and practitioners in both the current and future pandemics to identify more vulnerable groups and target prevention initiatives.

## Introduction

Migrants and ethnic minorities are reported to be at increased risk of acquiring COVID-19 and to have worse clinical outcomes of COVID-19 compared with the majority population.^1–5^ National statistics from Denmark and other Scandinavian countries correspondingly show that the incidence of COVID-19 cases is higher among individuals of non-Western origin, particularly of Middle Eastern origin, compared with individuals of Danish and other Western origin.^6^^,^^7^ In 2020, individuals of non-Western origin made up 58% of immigrants in Denmark, and among descendants, 83% were of non-Western origin.^8^ Therefore, the focus of this study is on individuals of non-Western origin, as they make up the majority of immigrants and have a higher incidence of COVID-19 than individuals of Western origin.

Research on previous pandemics, including the 1918 Influenza and the 2009 A/H1N1 Influenza, likewise shows immigrants and ethnic minorities as disproportionately affected compared with majority populations.^9^^,^^10^ This is hypothetically explained by various factors related to socioeconomy, language barriers, cultural norms and comorbidity in combination with poorer access to healthcare.^9–11^ First, lower income among immigrants and ethnic minorities may result in more people living together in closer proximity: data from Denmark show that the proportion of COVID-19 cases of non-Western origin more often live ≥5 individuals at the same address compared with ethnic Danes and individuals of Western origin.^6^ Furthermore, some immigrants and ethnic minority groups may have a culture of multi-generational living, which challenges social distancing and exposes older individuals with comorbidities to be at higher risk of COVID-19.^11^ In addition, multi-generational living may as well be a function of socioeconomic disadvantages besides being a cultural phenomenon.^12^ Second, many immigrants and ethnic minorities are more vulnerable to cardiovascular disease, diabetes and hypertension, which are all associated with more severe COVID-19.^11^^,^^13–15^ Third, compared to ethnic Danes, individuals of non-Western origin are more likely to work ‘frontline’ jobs within, e.g. transportation, hospitality, cleaning and operational services, and social welfare institutions; these jobs typically preclude working from home and are associated with a higher prevalence of COVID-19 across populations.^6^^,^^16^ Finally, language barriers may account for higher vulnerability among immigrants and ethnic minorities if, for example, guidelines on COVID-19 from the national and regional health authorities are not adequately translated and communicated.

These associations all make immigrants and ethnic minorities more vulnerable to COVID-19. Some of these associations have already been documented in reports and scientific papers from England and the USA.^3–5^^,^^17^ However, further studies are needed from other European immigration countries, which have different compositions of immigrants and ethnic minorities as well other contextual factors at play. Furthermore, adults of non-Western origin have a higher morbidity, but a lower mortality compared with individuals of Danish origin.^18^ Yet, no national health initiatives have been implemented in order to promote the health status of individuals of non-Western origin.^18^ Moreover, large-scale register-based studies are needed to disentangle the contribution of socioeconomic, demographic and comorbidity factors on COVID-19 hospitalization. Therefore, the aim of this study is to analyse the contribution of different socioeconomic and demographic risk factors, including occupational status, income, education, household size and population density, on hospitalizations for COVID-19 among immigrants and ethnic minorities compared to the majority population.

## Methods

### Study population

The study population is based on individuals residing in Denmark aged >17 years on 1 January 2020. Cases are identified by being registered as inpatients (admitted for ≥24 h) with a COVID-19 diagnosis of B34.2, B34.2A, B97.2 or B97.2A according to the 10th version of International Classification of Diseases (ICD-10) registered between 1 February 2020 and 30 June 2020 (*N* = 2547). COVID-19 cases are defined as being diagnosed with COVID-19 as the primary reason for hospital admission. Controls are formed by a random control sample of 500 000 individuals in the general population aged >17 years. Individuals who do not have information on sex or birthdate, or are aged <18 years (*N* = 81), have a COVID-19 diagnosis as the secondary reason for hospital contact (*N* = 308), or have negative values of equivalized disposable income (*N* = 1522) are excluded. COVID-19 cases identified in the random controls sample are recoded as cases instead of controls (*N* = 287). The final study population consists of 500 349 individuals including 2232 cases and 498 117 controls (Supplementary figure S1).

### Covariates

#### Country of birth

The study population is categorized using country of birth (Danish/Immigrant), or mother’s country of birth (Descendant) as a proxy hereof, to define individuals’ ethnicity. Ethnicity is defined in three ways as: (i) origin categorized as Danish (a person of Danish origin has at least one parent, who is a Danish citizen born in Denmark), immigrant (an immigrant is born in a foreign country and none of the parents are Danish citizens born in Denmark), or descendant (a descendant is born in Denmark and none of the parents are Danish citizens born in Denmark); (ii) grouped country of birth categorized as Danish (Denmark), Western (all EU countries, Andorra, Australia, Canada, Iceland, Liechtenstein, Monaco, New Zealand, Norway, San Marino, Switzerland, USA or the Vatican) and non-Western (Albania, Bosnia and Herzegovina, Belarus, Kosovo, North Macedonia, Moldova, Montenegro, Russia, Serbia, the Soviet Union, Turkey, Ukraine, former Yugoslavia, all countries in Africa, South- and Central America, Asia, Oceania with the exception of Australia and New Zealand, or stateless)^19^; and (iii) seven specific countries of birth, where a minimum of 10 COVID-19 cases are identified to compare with individuals born in Denmark (Denmark, Iraq, Lebanon, Morocco, Pakistan, Somalia, Turkey or former Yugoslavia). Information on country of birth is obtained from Statistics Denmark.^20^ For the descriptive analyses, immigrants and descendants are separated, and for the regression analyses, immigrants and descendants are considered as one group. Individuals are categorized into Western and non-Western countries of birth without distinguishing between being immigrant or descendant. The length of residence is also included for immigrants as a proxy for integration with a range from 0 to 34 years of residence and was categorized as ≤5; 6–15; 16–29; ≥30 years.

#### COVID-19

National register-based hospital data on cases of COVID-19 are obtained from The National Patient Register, which includes information on diagnoses whenever an individual is in contact with any hospital department by inpatient, outpatient, or emergency room visits.^21^ Data are obtained on all individuals registered with the following ICD-10 COVID-19 diagnoses: coronavirus infection, unspecified site (ICD-10: B34.2; B34.2A), or coronavirus as the cause of diseases classified to other chapters (ICD-10: B97.2, B97.2A).^22^ To increase the validity of COVID-19 diagnoses, COVID-19 cases are defined as inpatients hospitalized for at least 24 h and only with COVID-19 as the primary reason for hospitalization.

#### Socioeconomic and demographic factors

Data on socioeconomic and demographic factors are obtained from Statistics Denmark.^20^ The covariates include: (i) marital status: unmarried vs. married; (ii) highest-attained educational level based on International Standard Classification of Education (ISCED)^23^: low (primary school; ISCED levels: 0–2); medium (upper secondary education, business high school, vocational education; ISCED levels: 3–4); and high (short-term further education, middle-range education, bachelor’s, extended education, research degree; ISCED levels: 5–8); (iii) persons per household: range: 1 to ≥5 persons; (iv) occupation defined by either business industry or occupational status for those, who are unemployed or with unknown business industry: (a) business industry: farming, forestry and fishery; manufacture, raw material extraction and supply company; building and construction; trade and transportation; information and communication; financing and insurance; property trading and rental service; business service; public administration, education and health and social services; culture, leisure and other services; and (b) employed with unknown business industry (self-employed, collaborating spouse, salary earner); other occupational inactivity (retired persons, other economically inactive persons, who have no predominant income and welfare benefits are lower than the basic amount); and unemployed (unemployed person, unemployment benefit claimant, welfare claimant); (v) equivalized disposable income level based on household per year in Danish Kroner (DKK) divided into quartiles (Q1: ≤178 169 DKK, Q2: 178 170–246 364 DKK, Q3: 246 365–334 495 DKK, Q4: >334 495 DKK) and (vi) population density calculated by number of total individuals in a province divided by area in km^2^ ranging from 60 to 4340 persons/km^2^ and categorized based on quartiles into: <83 persons/km^2^, 83–310 persons/km^2^, ≥310 persons/km^2^.

#### Comorbidity

To understand the contribution of comorbidity on the OR of COVID-19 hospitalization among immigrants and ethnic minorities compared to the majority population, information is obtained on all ICD-10 diagnoses from The National Patient Register registered between 1980 and 2020.^21^ Comorbidities are weighted by the Charlson Comorbidity Index (CCI) to create a continuous weighted comorbidity score.^24^^,^^25^ The comorbidity scores assign weights from 1–6 to each comorbidity for every individual, which then receives a sum of weights based on all present comorbidities in an individual. A comorbidity score of 0 represents no comorbidities; higher scores indicate more severe comorbidities. The CCI is categorized into a score of: 0, 1, 2 or ≥3.

### Statistical analyses

Descriptive analyses are conducted to investigate the distribution of comorbidity, socioeconomic and demographic factors among individuals with and without COVID-19.

For the main analyses, logistic regression models are used to calculate odds ratios (OR) of COVID-19 hospitalization and 95% confidence intervals (95% CI) among different ethnic groups. Different categorizations of ethnic groups are used to investigate whether the OR of hospitalization with COVID-19 differed depending on the definition of ethnicity. First, the OR of hospitalization with COVID-19 is investigated among individuals of immigrants and descendants, Western origin, non-Western origin and residence length of immigrants compared with individuals of Danish origin. Nine models are applied to adjust for several covariates, including age, sex, CCI, household size, occupation, population density, equivalized disposable income, and educational level. Further, based on the ethnic groups, who are most frequently hospitalized with COVID-19, the OR of hospitalization with COVID-19 is investigated among individuals of specific ethnic origins compared with Danish origin. In sensitivity analyses, it is investigated whether individuals of non-Western origin, compared with individuals of Danish origin, are at higher risk of hospitalization with COVID-19 by including interaction terms between origin and either occupational group (farming, forestry, fishery, manufacture, raw material extraction and supply company, business service, trade and transportation, public administration, education and social and health services, other occupational inactivity, unemployed), or household size (1 to ≥5 persons), respectively. In additional analyses, the OR of hospitalization with COVID-19 is investigated for different risk factors presented as unadjusted and adjusted OR estimates.

SAS Enterprise Guide version 7.1 is used to conduct all analyses including a statistical significance level of 0.05. The study is approved by the University of Copenhagen Ethics Board (reference number: 514-0231/18-3000). Individual consent is not required by Danish legislation for register-based studies.

## Results

In this national register-based study, 2232 individuals are hospitalized due to COVID-19 in Denmark between February and June 2020. Table  1 shows the descriptive analyses. The median age at hospitalization with COVID-19 is 67 years with an overall equal distribution of men and women. More than 80% of hospitalized COVID-19 cases are of Danish origin. Yet, a higher number of COVID-19 cases per 10 000 persons are of non-Western origin or other origins than Danish. Out of the 2232 COVID-19 cases, 13 individuals (0.6%) are from the Nordic countries Finland, Norway and Sweden. Among immigrants, there is a higher number of COVID-19 cases where the individual’s length of residence exceeds 30 years. Having a higher CCI shows a higher number of COVID-19 cases per 10 000 persons. The COVID-19 cases are not observed specifically in some business industries, but a higher proportion of COVID-19 cases is occupationally inactive, thus, either retired or economically inactive. There is also a higher number of COVID-19 cases in the provinces around Copenhagen.

**Table 1:** Baseline characteristics of COVID-19 cases in the study population (*N* = 500 349) and percentage of the Danish adult population (*N* = 4 671 587)

		Hospitalized COVID-19 cases in Denmark, *N* = 2232	Total in study population, *N* = 500 349	No. of hospitalized COVID-19 cases per 10 000 persons,^a^*N* = 4 671 587
Characteristics		No. (%)	No.	No.
Age group, no. (%),	<30 years	114 (5%)	91 083	1
*N* = 500 349	30–39 years	140 (6%)	74 213	1
	40–49 years	265 (12%)	79 475	3
	50–59 years	336 (15%)	86 086	3
	60–69 years	359 (16%)	72 212	4
	≥70 years	1018 (46%)	97 280	9
Sex, no. (%), *N* = 500 349	Women	1027 (46%)	253 755	3
Origin, no. (%),	Danish	1893 (85%)	431 646	4
*N* = 500 349	Immigrant	313 (14%)	59 801	4
	Descendant	26 (1%)	8902	2
Grouped country of birth, no. (%), *N* = 500 349	Danish	1893 (85%)	431 646	4
	Western^b^	63 (3%)	26 270	2
	Non-Western	276 (12%)	42 433	6
	Immigrant	253 (92%)	34 736	6
	Descendant	23 (8%)	7697	2
Country of birth, no. (%),	Denmark	1893 (92%)	431 646	4
*N* = 448 001	Iraq	35 (2%)	2678	12
	Lebanon	13 (1%)	2163	5
	Morocco	21 (1%)	971	20
	Pakistan	30 (1%)	2134	13
	Somalia	23 (1%)	1387	15
	Turkey	35 (2%)	5697	5
	Former Yugoslavia	11 (0.5%)	1325	7
Length of residence for immigrants, no. (%), *N* = 59 801	≤5 years	22 (7%)	19 849	1
	6–15 years	42 (13%)	13 283	2
	16–29 years	104 (33%)	15 592	6
	≥30 years	145 (46%)	11 077	12
Marital status, no. (%), *N* = 500 349	Unmarried	1053 (47%)	270 235	3
Educational level, no. (%),	Low	619 (28%)	121 030	4
*N* = 493 820^c^	Medium	864 (40%)	206 914	3
	High	692 (32%)	164 384	3
Charlson Comorbidity Index, no. (%), *N* = 500 349	0	1330 (62%)	434 589	2
	1	317 (15%)	30 589	9
	2	259 (12%)	22 584	10
	≥3	247 (11%)	12 508	18
No. of persons per household, no. (%), *N* = 500 349	1 person	715 (32%)	11 212	5
	2 persons	895 (40%)	188 842	4
	3 persons	245 (11%)	77 918	2
	4 persons	210 (9%)	74 819	2
	≥5 persons	167 (7%)	45 558	3
Occupation, no. (%),	Unemployed	32 (1%)	9992	2
*N* = 496 819^d^	Employed without known business industry	46 (2%)	9427	4
	Other occupational inactivity	1050 (47%)	120 252	8
	Farming, forestry, fishery, manufacture, raw material extraction and supply company^e^	108 (5%)	46 183	2
	Building and construction	58 (3%)	20 227	2
	Trade and transportation	209 (9%)	85 982	2
	Information and communication	46 (2%)	13 584	3
	Financing and insurance	34 (1%)	10 158	3
	Property trading and rental services	21 (1%)	5744	3
	Business service	130 (6%)	41 984	2
	Public administration, education and health and social services	453 (20%)	116 694	3
	Culture, leisure and other services	42 (2%)	17 381	2
Province, no. (%),	Copenhagen city (PD^f^** **=** **4340 persons/km^2^)	513 (23%)	66 553	7
*N* = 500 349	Copenhagen area (PD** **=** **1602 persons/km^2^)	335 (15%)	34 699	9
	North Zealand (PD** **=** **320 persons/km^2^)	348 (16%)	49 825	6
	East Zealand (PD** **=** **310 persons/km^2^)	103 (4%)	21 671	4
	West- and South Zealand (PD** **=** **91 persons/km^2^)	290 (13%)	49 518	5
	Funen (PD** **=** **143 persons/km^2^)	103 (4%)	43 077	2
	North Jutland (PD** **=** **75 persons/km^2^)	124 (6%)	53 037	2
	East Jutland (PD** **=** **154 persons/km^2^)	101 (4%)	77 786	1
	West Jutland (PD** **=** **60 persons/km^2^)	152 (7%)	37 032	3
	South Jutland (PD** **=** **82 persons/km^2^)	153 (7%)	63 483	2
	Bornholm (PD** **=** **67 persons/km^2^)	10 (1%)	3668	2
Income, no. (%),	1st quartile (≤178 169 DKK)	594 (27%)	128 540	4
*N* = 500 349	2nd quartile (178 170–246 364 DKK)	594 (27%)	123 904	4
	3rd quartile (246 365–334 495 DKK)	457 (20%)	123 945	3
	4th quartile (>334 495 DKK)	587 (26%)	123 960	4

a The number of hospitalized COVID-19 cases per 10 000 persons in the Danish population. Example: Among women, there are three hospitalized COVID-19 cases per 10 000 persons in the Danish population. Weight is calculated by: (COVID-19 cases/Total number of individuals in respective group)/10.7)*100.

b There are too few COVID-19 cases to be displayed among immigrants and descendants of Western origin.

c There are 6529 individuals without information on educational level.

d There are 139 671 individuals without information on business industry, but with information on occupational status (unemployed/employed without known business industry/other occupational inactivity) and 3530 individuals without information on neither business industry or occupational status.

e Business industries of farming, forestry, fishery and manufacture, raw material extraction and supply company are combined into one group due to few cases in some industries.

f PD: population density.

The results of the main analyses (table 2) show that among all immigrants and descendants compared with Danes, there is a 13% higher OR of hospitalization with COVID-19 adjusting for sex, a 79% higher OR (95% CI: 1.56–2.01) adjusting for age and a 37% higher OR (95% CI: 1.19–1.56) adjusting further for CCI and all socioeconomic and demographic factors.

**Table 2:** Odds ratio (OR) with 95% confidence intervals (95% CI) of hospitalization with COVID-19 among individuals grouped by regional origin, immigrants and descendants, and specific countries of birth living in Denmark, *N* = 500 349

			Crude	Sex	+ Age	+ CCI	+ Household size	+ Population density	+ Income	+ Occupation	+ Educational level
Group	No. of individuals	COVID-19 cases	OR (95% CI)	OR (95% CI)	OR (95% CI)	OR (95% CI)	OR (95% CI)	OR (95% CI)	OR (95% CI)	OR (95% CI)	OR (95% CI)
Danish origin (ref.)	431 646	1893	1.00	1.00	1.00	1.00	1.00	1.00	1.00	1.00	1.00
Immigrants and descendants	68 703	339	1.13 (1.00–1.26)	1.13 (1·00–1.26)	1.79 (1.56–2.01)	1.76 (1.55–1.98)	1.65 (1.46–1.87)	1.35 (1.19–1.53)	1.36 (1.19–1.54)	1.39 (1.22–1.59)	1.37 (1.19–1.56)
Danish origin (ref.)	431 646	1893	1.00	1.00	1.00	1.00	1.00	1.00	1.00	1.00	1.00
Western origin	26 270	63	0.55 (0.43–0.70)	0.54 (0.42–0.70)	0.79 (0.62–1.02)	0.80 (0.62–1.03)	0.78 (0.60–1.01)	0.67 (0.52–0.86)	0.67 (0.52–0.87)	0.68 (0.52–0.88)	0.67 (0.51–0.88)
Non-Western origin	42 433	276	1.49 (1.31–1.69)	1.49 (1.31–1.69)	2.54 (2.23–2.89)	2.44 (2.14–2.79)	2.28 (1.99–2.61)	1.81 (1.58–2.08)	1.85 (1.60–2.14)	1.91 (1.65–2.21)	1.87 (1.60–2.17)
Denmark (ref.)	431 646	1893	1.00	1.00	1.00	1.00	1.00	1.00	1.00	1.00	1.00
Iraq^a^	2678	35	3.01 (2.15–4.21)	2.97 (2.12–4.16)	4.79 (3.41–6.73)	4.77 (3.38–6.73)	4.44 (3.14–6.29)	3.52 (2.49–5.00)	3.62 (2.54–5.15)	3.77 (2.64–5.37)	3.61 (2.45–5.33)
Lebanon^a^	2163	13	1.37 (0.79–2.37)	1.36 (0.79–2.35)	2.48 (1.43–4.29)	2.49 (1.44–4.32)	2.32 (1.34–4.03)	2.07 (1.19–3.59)	2.13 (1.22–3.71)	2.18 (1.25–3.81)	2.20 (1.23–3.92)
Morocco^a^	971	21	5.02 (3.25–7.75)	5.00 (3.24–7.73)	7.63 (4.92–11.84)	7.58 (4.88–11.78)	7.17 (4.61–11.15)	4.53 (2.91–7.06)	4.69 (2.99–7.34)	4.76 (3.04–7.46)	3.70 (2.19–6.26)
Pakistan^a^	2134	30	3.24 (2.25–4.66)	3.21 (2.23–4.62)	5.06 (3.51–7.29)	4.70 (3.25–6.79)	4.19 (2.88–6.10)	2.50 (1.72–3.65)	2.58 (1.76–3.78)	2.68 (1.83–3.93)	2.81 (1.90–4.14)
Somalia^a^	1387	23	3.83 (2.53–5.80)	3.81 (2.52–5.77)	7.24 (4.76–11.01)	6.45 (4.16–10.00)	5.92 (3.80–9.21)	5.36 (3.44–8.36)	5.50 (3.51–8.62)	5.88 (3.74–9.25)	5.88 (3.62–9.57)
Turkey^a^	5697	35	1.40 (1.00–1.96)	1.40 (1.00–1.95)	2.33 (1.66–3.27)	2.28 (1.63–3.20)	2.12 (1.51–2.99)	1.55 (1.10–2.19)	1.61 (1.14–2.27)	1.65 (1.16–2.33)	1.63 (1.13–2.34)
Former Yugoslavia^a^	1325	11	1.90 (1.05–3.45)	1.90 (1.05–3.45)	2.45 (1.35–4.45)	1.92 (0.99–3.72)	1.81 (0.93–3.50)	1.27 (0.66–2.46)	1.30 (0.67–2.53)	1.33 (0.68–2.58)	1.44 (0.74–2.79)
Residence length +30 years (ref.)^b^	11 077	145	1.00	1.00	1.00	1.00	1.00	1.00	1.00	1.00	1.00
Residence length 16–29 years^b^	15 592	104	0.51 (0.39–0.65)	0.51 (0.40–0.66)	0.93 (0.70–1.23)	0.94 (0.70–1.25)	0.89 (0.67–1.19)	1.04 (0.78–1.40)	1.03 (0.76–1.38)	1.02 (0.76–1.38)	1.18 (0.85–1.64)
Residence length 6–15 years^b^	13 283	42	0.24 (0.17–0.34)	0.24 (0.17–0.34)	0.63 (0.42–0.94)	0.66 (0.44–1.00)	0.65 (0.43–0.98)	0.80 (0.53–1.22)	0.80 (0.53–1.21)	0.85 (0.56–1.30)	0.88 (0.55–1.40)
Residence length <5 years^b^	19 849	22	0.08 (0.05–0.13)	0.08 (0.05–0.13)	0.28 (0.17–0.47)	0.31 (0.18–0.52)	0.31 (0.18–0.52)	0.38 (0.22–0.64)	0.36 (0.21–0.61)	0.42 (0.24–0.74)	0.36 (0.19–0.70)

a The analyses on specific countries of birth are based on 448 001 individuals.

b The analyses on immigrants’ residence length are based on 59 801 individuals.

Among individuals of Western origin compared with individuals of Danish origin, there is a 46% lower OR of hospitalization with COVID-19 (95% CI: 0.42–0.70) adjusting for sex, which changes to a 21% lower OR (95% CI: 0.62–1.02) adjusting for age, and a 33% lower OR (95% CI: 0.51–0.88) adjusting for CCI and socioeconomic and demographic factors. Compared with individuals of Danish origin, individuals of non-Western origin have a 49% higher OR of hospitalization with COVID-19 (95% CI: 1.31–1.69) adjusting for sex, a 2.54 times higher OR (95% CI: 2.23–2.89) adjusting for age and an 87% higher OR (95% CI: 1.60–2.17) adjusting for CCI and all socioeconomic and demographic factors.

The results of the analyses for specific countries show that the OR of hospitalization with COVID-19 is highest among individuals from Somalia, followed by Morocco, Iraq, Pakistan, Lebanon, Turkey and former Yugoslavia.

Overall, the results of the analyses on immigrants’ residence length show that when comparing with immigrants who lived in Denmark for ≥30 years, the risk of COVID-19 hospitalization is lowest among immigrants who lived in Denmark ≤5 years, while the risk is higher among immigrants who lived in Denmark for 16–29 years. However, the results are not statistically significant for immigrants, who lived in Denmark between 16–29 years.

For the sensitivity analyses, specific groups of business industries or occupational activity are selected based on having prevalent COVID-19 cases among individuals of non-Western origin, and the analyses are grouped by these occupational groups using individuals of Danish origin as reference group (table 3). The results show that for all occupational groups, individuals of non-Western origin have a higher OR of hospitalization with COVID-19 in a range of ORs from 1.27–3.01 compared with individuals of Danish origin. In the analyses for household size, the OR of hospitalization with COVID-19 is investigated in individuals of non-Western origin compared with individuals of Danish origin (table 3). Among individuals of non-Western origin, the ORs of hospitalization with COVID-19 are observed to be 1.52–2.37 times higher compared with individuals of Danish origin.

**Table 3:** Odds ratio (OR) with 95% confidence intervals (95% CI) of hospitalization with COVID-19 among individuals of non-Western origin compared with Danish origin grouped by specific occupational groups in Denmark with prevalent COVID-19 cases and by number of persons in households

				Crude			Sex	+ Age	+ CCI	+ Household size	+ Population density	+ Income	+ Educational level
		No. of individuals	COVID-19 cases	OR (95% CI)			OR (95% CI)	OR (95% CI)	OR (95% CI)	OR (95% CI)	OR (95% CI)	OR (95% CI)	OR (95% CI)
Origin and occupation, *N* = 399 980													
Danish origin (ref.)		361 489	1671	1.00			1.00	1.00	1.00	1.00	1.00	1.00	1.00
Non-Western origin		36 565	255										
	Farming and manufacture^a^	3310	17	2.27 (1.35–3.82)			2.30 (1.37–3.87)	3.38 (2.00–5.69)	3.48 (2.06–5.87)	3.28 (1.94–5.54)	2.88 (1.71–4.87)	2.89 (1.71–4.90)	3.01 (1.77–5.09)
	Business service	5337	22	1.34 (0.85–2.13)			1.38 (0.87–2.19)	1.86 (1.17–2.95)	1.72 (1.07–2.76)	1.62 (1.01–2.59)	1.34 (0.83–2.15)	1.35 (0.84–2.17)	1.27 (0.77–2.09)
	Trade and transportation	9507	43	1.95 (1.40–2.74)			1.91 (1.36–2.67)	2.56 (1.83–3.60)	2.55 (1.81–3.59)	2.35 (1.66–3.31)	1.78 (1.26–2.51)	1.77 (1.25–2.51)	1.77 (1.24–2.53)
	Public administration, education and social and health services	7283	57	2.14 (1.62–2.83)			2.16 (1.63–2.85)	3.00 (2.26–3.97)	2.96 (2.23–3.93)	2.80 (2.11–3.72)	2.19 (1.65–2.91)	2.19 (1.65–2.92)	2.20 (1.65–2.94)
	Other occupational inactivity	8170	98	1.39 (1.12–1.71)			1.39 (1.12–1.71)	2.72 (2.19–3.37)	2.62 (2.10–3.27)	2.50 (2.00–3.13)	1.92 (1.53–2.40)	1.88 (1.49–2.36)	1.82 (1.42–2.33)
	Unemployed	2958	18	3.44 (1.66–7.16)			3.56 (1.71–7.40)	2.87 (1.38–5.97)	3.57 (1.65–7.76)	3.30 (1.52–7.18)	2.95 (1.35–6.41)	2.93 (1.35–6.39)	2.30 (0.95–5.59)
Origin and number of persons in households, *N* = 474 079													
Danish origin (ref.)		431 646	1893		1.00	1.00		1.00	1.00	1.00	1.00	1.00	1.00
Non-Western origin		42 157	276										
	1 person	5141	37		1.14 (0.81–1.58)	1.11 (0.80–1.55)		1.94 (1.39–2.71)	1.86 (1.32–2.63)	1.46 (1.03–2.06)	1.46 (1.03–2.06)	1.56 (1.10–2.22)	1.52 (1.04–2.22)
	2 persons	9608	69		1.51 (1.18–1.93)	1.52 (1.19–1.95)		2.56 (2.00–3.28)	2.46 (1.90–3.17)	1.90 (1.47–2.45)	1.94 (1.50–2.51)	2.04 (1.57–2.66)	2.02 (1.53–2.68)
	3 persons	8578	60		2.51 (1.87–3.36)	2.52 (1.88–3.38)		2.86 (2.13–3.83)	2.75 (2.05–3.71)	2.29 (1.70–3.09)	2.37 (1.75–3.20)	2.61 (1.90–3.60)	2.37 (1.69–3.33)
	4 persons	8308	43		1.98 (1.42–2.78)	2.00 (1.43–2.80)		2.07 (1.48–2.90)	1.93 (1.37–2.72)	1.59 (1.13–2.24)	1.65 (1.17–2.33)	1.75 (1.21–2.53)	1.84 (1.26–2.67)
	≥5 persons	10 522	67		2.15 (1.57–2.95)	2.15 (1.57–2.95)		2.37 (1.73–3.25)	2.26 (1.64–3.12)	1.78 (1.29–2.45)	1.81 (1.31–2.52)	1.84 (1.29–2.63)	1.79 (1.24–2.59)

a Farming, forestry, fishery, manufacture, raw material extraction and supply company are abbreviated as Farming and manufacture due to limited space in this table.

In the adjusted analyses where different risk factors are investigated separately in relation to the OR of hospitalization with COVID-19 (table 4), the highest OR’s are observed in individuals of non-Western origin, the older age groups, those with higher CCI, those living five or more individuals together in one household, those working within public administration, education and health and social services, and those who are occupationally inactive.

**Table 4:** Unadjusted and adjusted odds ratio (OR) with 95% confidence intervals (95% CI) of hospitalization with COVID-19 by risk factor, *N* = 500 349

				Unadjusted (*N* = 500 349)	Adjusted^a^ (*N* = 489 781)
Risk factor		No. of individuals	COVID-19 cases	OR (95% CI)	OR (95% CI)
Grouped country of	Danish	431 646	1893	1.00	1.00
birth	Western	26 270	63	0.55 (0.43–0.70)	0.66 (0.50–0.87)
	Non-Western	42 433	276	1.49 (1.31–1.69)	1.80 (1.55–2.10)
Sex	Men	246 594	1205	1.00	1.00
	Women	253 755	1027	0.83 (0.76–0.90)	0.75 (0.68–0.82)
Age groups	<30 years	91 083	114	1.00	1.00
	30–39 years	74 213	140	1.51 (1.18–1.93)	1.39 (1.08–1.80)
	40–49 years	79 475	265	2.67 (2.14–3.33)	2.40 (1.91–3.03)
	50–59 years	86 086	336	3.13 (2.53–3.87)	2.70 (2.16–3.38)
	60–69 years	72 212	359	3.99 (3.23–4.92)	3.15 (2.50–3.97)
	≥70 years	97 280	1018	8.44 (6.95–10.24)	5.58 (4.40–7.07)
Charlson Comorbidity	0	434 589	1330	1.00	1.00
Index	1	30 589	317	3.41 (3.02–3.86)	2.33 (2.04–2.65)
	2	22 584	259	3.78 (3.31–4.32)	2.30 (2.00–2.65)
	≥3	12 508	247	6.56 (5.72–7.53)	3.67 (3.17–4.25)
Household size	1 person	113 212	715	1.00	1.00
	2 persons	188 842	895	0.75 (0.68–0.83)	0.84 (0.76–0.94)
	3 persons	77 918	245	0.50 (0.43–0.57)	0.96 (0.81–1.13)
	4 persons	74 819	210	0.44 (0.38–0.52)	0.97 (0.81–1.17)
	≥5 persons	45 558	167	0.58 (0.49–0.69)	1.12 (0.92–1.37)
Population density	>300 km^2^	170 381	494	1.00	1.00
	83–310 km^2^	157 220	439	0.38 (0.35–0.43)	0.37 (0.33–0.41)
	<83 km^2^	172 748	1299	0.37 (0.33–0.41)	0.36 (0.32–0.40)
Income	1st quartile (≤178 169 DKK)	128 540	594	0.98 (0.87–1.09)	0.84 (0.73–0.97)
	2nd quartile (178 170–246 364 DKK)	123 904	594	1.01 (0.90–1.14)	0.79 (0.69–0.90)
	3rd quartile (246 365–334 495 DKK)	123 945	457	0.78 (0.69–0.88)	0.80 (0.70–0.91)
	4th quartile (>334 495 DKK)	123 960	587	1.00	1.00
Occupation	Unemployed	9992	32	1.00	1.00
	Employed	9427	46	1.53 (0.97–2.40)	1.66 (0.97–2.83)
	Other occupational inactivity	120 252	1050	2.74 (1.93–3.90)	2.03 (1.29–3.18)
	Farming, forestry, fishery, manufacture, raw material extraction and supply company^b^	46 067	108	0.73 (0.49–1.09)	1.40 (0.87–2.26)
	Building and construction	20 160	58	0.90 (0.58–1.38)	1.60 (0.96–2.68)
	Trade and transportation	85 759	209	0.76 (0.52–1.10)	1.42 (0.90–2.25)
	Information and communication	13 556	46	1.06 (0.67–1.67)	1.49 (0.87–2.54)
	Financing and insurance	10 130	34	1.05 (0.65–1.70)	1.44 (0.83–2.52)
	Property trading and rental services	5730	21	1.15 (0.66–1.99)	1.42 (0.76–2.64)
	Business service	41 874	130	0.97 (0.66–1.43)	1.55 (0.97–2.49)
	Public administration, education and health and social services	116 530	453	1.22 (0.85–1.74)	2.17 (1.38–3.40)
	Culture, leisure and other services	17 342	42	0.76 (0.48–1.20)	1.27 (0.74–2.16)
Education	Low	121 030	619	1.22 (1.09–1.36)	1.09 (0.96–1.23)
	Medium	206 914	864	0.99 (0.90–1.10)	1.02 (0.92–1.14)
	High	164 384	692	1.00	1.00

a Adjusted for sex, age, CCI, household size, occupation, population density, income and educational level.

b Business industries of farming, forestry, fishery and manufacture, raw material extraction and supply company are combined into one group due to few cases in some industries.

## Discussion

Using an exceptional nationwide cohort, findings of this study provide unique information on the contribution of socioeconomic and demographic factors to the higher COVID-19 hospitalization risk among immigrants and ethnic minorities compared to ethnic Danes. Immigrants and descendants of non-Western origin have a 2.5 times higher OR of hospitalization. About half of the excess risk is explained by comorbidity, socioeconomic and demographic factors.

This study complements emerging evidence from other European countries that immigrants and ethnic minorities are at higher risk of hospitalization for COVID-19.^2^^,^^26^^,^^27^ The higher risk of COVID-19 hospitalization should be seen in the context of the higher positive incidence and mortality rates for COVID-19 among immigrants and ethnic minorities in Denmark and other European countries.^2^ Interestingly, existing literature shows that ethnic disparities in COVID-19 incidence, mortality and hospitalizations seem to cut across European countries with different compositions of immigrant and ethnic minority populations, ranging from the UK and France, with many minorities originating from former colonies, to ‘new’ immigration countries like the Nordics, where labour immigrants, refugee populations and family-reunified individuals comprise the vast proportion of non-Western immigrants.^28^ This pattern indicates that cross-cutting factors related to socioeconomic vulnerability are at stake. First, ethnic minorities may live together in larger households facilitating transmission and impeding isolation, possibly due to socioeconomic deprivation and cultural factors as multi-generational living. Data from Denmark observe that more families of non-Western origin live in households of fewer m^2^ per person in general, which also applies for those with a COVID-19 infection.^29^^,^^30^ In our study, about 50% of the hospitalized COVID-19 cases who live ≥5 persons per household are of Danish origin, while 40% are of non-Western origin, and the minority are of Western origin. Thus, cases of COVID-19 infections and hospitalizations, respectively, show different patterns in terms of ethnicity and household size, as we do not observe a dose-response relation between ORs and household size. Second, COVID-19 hospitalization among ethnic minorities seem to be related with occupational groups, where working from home is not a possibility, as observed in other research.^31^ Third, population density is an important factor to hospitalization risk, which is supported by a Swedish study, where neighbourhood population density together with income, employment and household members, explained COVID-19 mortality risk.^32^ Finally, education and income do not markedly change the estimates, which could be because occupation, population density and household size work as a proxy hereof. As comorbidity, socioeconomic and demographic factors only explain some of the risk, it can only be speculated about the remaining contributory factors. Some explanations could be language barriers,^33^ ‘newness’,^34^ limited health literacy^35^ and genetic and biological susceptibility,^36^ which may be driving mechanisms potentially interacting in complex, dynamic ways within groups of immigrants and ethnic minorities. The complex interplay between factors related to language barriers, health literacy, access in healthcare, migration background and changing migration patterns may as well explain why immigrants with shorter residence length are at lower risk of COVID-19 hospitalization, which was against our expectations, as residence length was considered a proxy for integration over the years.

The key strength of this study is its basis on unique nationwide data representing all hospitalized COVID-19 cases in Denmark from February to June 2020. Using national data made it possible to include a large study population and a random reference group, link different national registers and utilize retrospective and recent data on several comorbidity, socioeconomic and demographic confounders and link them to each individual. The validity of COVID-19 diagnoses is increased by only including cases, where COVID-19 is the primary reason for hospital contact.

This study also has several limitations. First, Denmark’s management of the pandemic through restrictive policies resulted in a comparatively limited number of hospitalizations during the ‘first wave’ limiting the sample size of specific country groups and reducing statistical power in some analyses. Second, adjusting for CCI does not markedly change the results, yet, another Danish study finds an increased risk of COVID-19 among individuals with one or more comorbidities of the CCI.^37^ However, using a comorbidity index constructed specifically for COVID-19 could be more relevant. Third, the study is limited by the availability of data of the basis for residence permit and missing data on business industry and educational level. Fourth, the study population only includes the most severe COVID-19 cases hospitalized for ≥24 h and is not representative of COVID-19 cases in the general population. Therefore, the findings of this study only apply to the most severe COVID-19 cases among immigrants and ethnic minorities.

In conclusion, there is a higher OR of hospitalization with COVID-19 among immigrants originating from non-Western countries compared to ethnic Danes. The excess risk is explained by socioeconomic and demographic factors. Further studies including qualitative approaches are needed to understand the underlying reasons for the observed disparities. The results also highlight immigrants and ethnic minorities as more vulnerable groups in terms of acquiring COVID-19, and that this tendency should be investigated among COVID-19 cases in the general population as well. This knowledge is crucial for health policymakers and practitioners in both the current and future pandemics to identify more vulnerable groups and target prevention initiatives.

## Supplementary data

Supplementary data are available at *EURPUB* online.

## Funding

This study was funded by University Hospital Hvidovre, University of Copenhagen and Statens Serum Institut.

*Conflicts of interest*: T.B. reports grants from Novo Nordisk Foundation, Simonsen Foundation, Lundbeck Foundation, Pfizer, Gilead, Kai Hansen Foundation, and Erik and Susanna Olesen’s Charitable Fund, and personal fees from GSK, Pfizer, Boehringer Ingelheim, Gilead, MSD, and Pentabase, outside the submitted work. All other authors declare no competing interests.

## Data availability

The data underlying this article cannot be shared publicly, as the data are only available to researchers of this article based on individual data approval from Statistics Denmark.

Key points

- Findings of this study show that immigrants and descendants of non-Western origin had a 2.5 times higher OR of hospitalization with COVID-19.
- The excess risk is explained by comorbidity as well as socioeconomic and demographic factors, especially household size, occupation and population density.
- This knowledge is crucial for health policymakers and practitioners in both the current and future pandemics to identify more vulnerable groups and target prevention initiatives.

## Supplementary Material

ckab186_Supplementary_Data
