# Supplementary material for: Socioeconomic and demographic risk factors in COVID-19 hospitalization among immigrants and ethnic minorities
Source: Eur J Public Health. 2021 Oct 27;32(2):302–10. doi: 10.1093/eurpub/ckab186 (PMC8586727; doi:10.1093/eurpub/ckab186)
Supplement: ckab186_Supplementary_Data [file ckab186_supplementary_data.docx]

**Supplementary material to:**

**Socio-economic and Demographic Risk Factors in COVID-19 Hospitalization among Migrants and Ethnic Minorities**


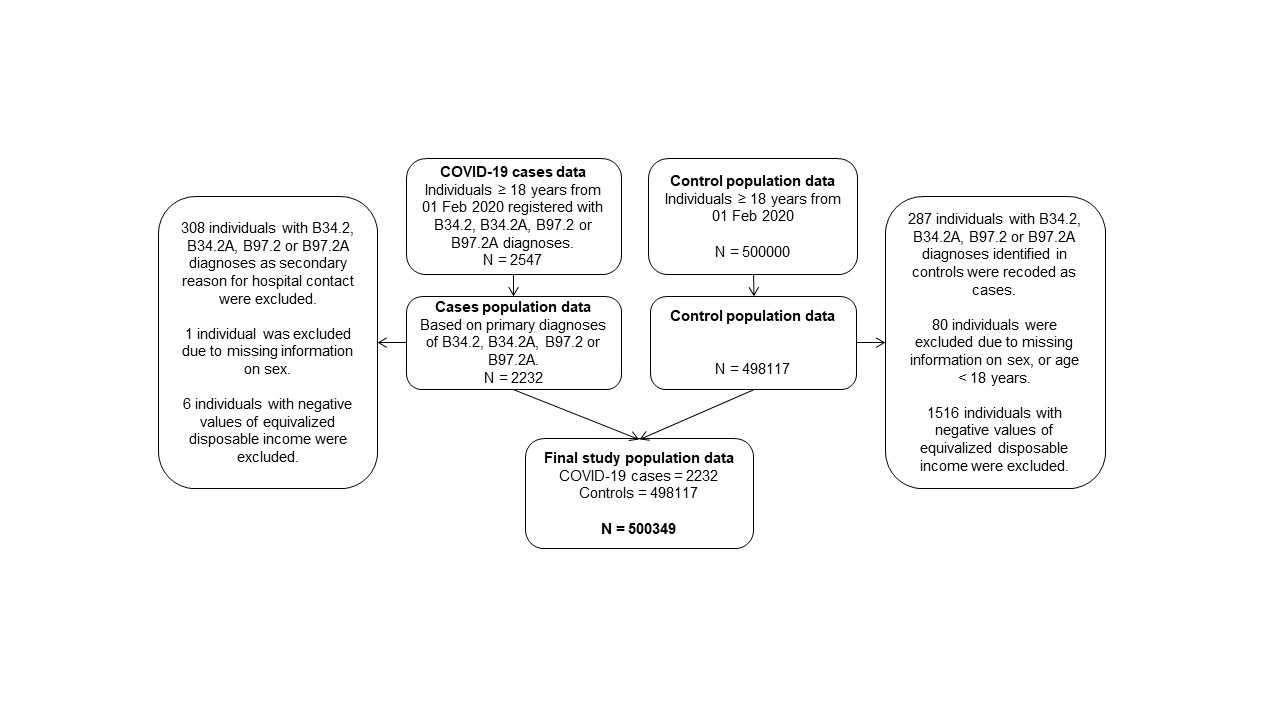


**Figure 1. Flowchart of the study population selected for analyses (N = 500349).**
